# Association of human immunodeficiency virus with acute myocardial infarction and presumed sudden cardiac death

**DOI:** 10.1016/j.resplu.2025.101035

**Published:** 2025-07-19

**Authors:** Patricia Jabre, Richard Chocron, Thomas Laurenceau, Marion Chabrol, Ugo Meli, Younès Youssfi, Marie Cécile Perier, Wulfran Bougouin, Frankie Beganton, Thomas Loeb, François Revaux, Daniel Jost, Alain Cariou, Jean-Philippe Empana, Frédéric Adnet, Xavier Jouven

**Affiliations:** a Service d’Aide Médicale d’Urgence—SAMU de Paris, Necker-Enfants malades Hospital, AP-HP, Paris, France; b Université Paris Cité, INSERM, UMR-S970, Paris Cardiovascular Research Center, Team “Integrative Epidemiology of Cardiovascular Diseases”, Paris, France; c Emergency Department, European Georges Pompidou Hospital, AP-HP, Paris, France; d Medical-Surgical Intensive Care Unit, Ramsay Générale de Santé, Jacques Cartier Private Hospital, Massy, France; e SAMU 92, Raymond Poincaré Hospital, AP-HP, Paris, France; f SAMU 94, Mondor Hospital, AP-HP, Créteil, France; g Brigade Sapeurs-Pompiers de Paris, Paris, France; h Medical Intensive Care Unit, Cochin Hospital, AP-HP, Paris, France; i Cardiology Department, Georges Pompidou European Hospital, AP-HP, Paris, France

**Keywords:** Human immunodeficiency virus, Acute myocardial infarction, Presumed sudden cardiac death

## Abstract

**Background:**

While extensive evidence linking human immunodeficiency virus (HIV) infection to acute myocardial infarction (AMI), several studies have also suggested an association between HIV and presumed sudden cardiac death (PSCD). Our objective was to evaluate the association between HIV and PSCD compared to AMI. Understanding whether HIV confers differential risks for distinct cardiovascular outcomes is essential to guide prevention strategies and risk stratification in this population.

**Methods:**

The study design was a case-control study. We combined data from the large prospective population-based Paris Sudden Death Expertise Center Registry on PSCD and from the French National Health Insurance (SNDS) database. The SNDS database contains comprehensive data on all reimbursements for health-related expenditures and detailed medical information on all admissions to French public and private hospitals. In this study, adult patients with PSCD that occurred between 2011 and 2020 in Paris and the 3 adjacent departments were matched with AMI controls. We identified HIV patients in the 2 populations. We used a logistic regression to estimate the association between HIV and PSCD compared to HIV and AMI, adjusted for confounders.

**Results:**

In this study, 22,510 PSCD patients (60% men, age 71 (17) years) were matched with 22,510 AMI controls (60% men, age 72 (17) years). Among them, 245 (1%) and 104 (1%) had a positive HIV status preceding PSCD and AMI respectively. The odds of PSCD was 97% higher than the odds of AMI in HIV patients (adjusted odds-ratio, 1.97; 95% confidence interval: 1.55–2.49)**.**

**Conclusions:**

Our findings, based on big data analysis, strongly suggest a significant association between HIV status and PSCD, also among patients without a history of AMI. The underlying mechanisms still remain incompletely defined and further studies are needed.

## Background

Human immunodeficiency virus (HIV) has gone from an acute and fatal disease to a chronic, non-fatal disease in a matter of years.^1^, ^2^ The effectiveness of treatments including antiretroviral therapy (ART) has enabled this rapid and beneficial change. However, infection with HIV has been shown to be associated with almost 50 % increased risk of acute myocardial infarction (AMI) beyond that explained by recognized risk factors.^3^, ^4^, ^5^, ^6^ This important concern has been extensively addressed. Accelerated atherosclerosis in individuals with HIV may result from a combination of factors, including pro-coagulant and pro-inflammatory mechanisms linked to immunosuppression; direct viral effects on endothelial and other cells; harmful metabolic side effects of certain ART agents, such as dyslipidaemia and insulin resistance; a high prevalence of smoking and substance use; and genetic predisposition.^7^, ^8^, ^9^, ^10^

As for presumed sudden cardiac death (PSCD), several studies have suggested a significant association with HIV.^11^, ^12^, ^13^, ^14^, ^15^, ^16^ The common point between studies that evaluated HIV associations with AMI and PSCD is that they compared HIV individuals to the general population with a small number of AMI and PSCD events.^17^, ^18^ To understand if the etiology behind PSCD among HIV patients differ from patients without HIV, we aimed to evaluate the association between HIV and PSCD compared to HIV and AMI. For this, we combined data from a large population-based registry on PSCD and the French National Health Insurance (SNDS) database.^19^

## Materials and methods

### Design and sources

The study design was a case-control study.

#### Paris sudden death expertise center (SDEC) registry

The Paris SDEC registry includes all patients with PSCD that occurred between 2011 and 2020 in Paris and its three adjacent departments which covers a population of 6.7 million—approximately 10 % of the French population. This Paris SDEC registry, previously described, provides a comprehensive, prospective, and continuous record of information on the occurrence (Utstein criteria), management (pre- and in-hospital) and patient outcomes (regarding survival and neurological outcome) of all PSCD cases.^20^, ^21^, ^22^ The review boards approved the SDEC registry (CNIL authorization DR-2012-445,2012). No informed consent was required.

#### French National Health Insurance (SNDS) database

The SNDS database covers the entire French population (67.4 million inhabitants). The SNDS database links the claims database of the French National Healthcare System (SNIIRAM) and the French National Hospitalization Database (Programme de Médicalisation des Systèmes d’Information, PMSI).^19^, ^23^ The SNDS database provides comprehensive information on all reimbursed health-related expenses, including medications and outpatient medical and nursing care prescribed or delivered by healthcare professionals, along with demographic data. It also provides detailed medical information on all admissions to French public and private hospitals, including dates of hospital admission and hospital discharge, discharge diagnosis coded according to the international classification of diseases, 10th revision (ICD-10), and drugs coded according to the Anatomical Therapeutic Chemical (ATC) classification during the hospital stay.^24^, ^25^

### Population and definitions

#### Sudden cardiac death ascertainment

**Fig. 1:**
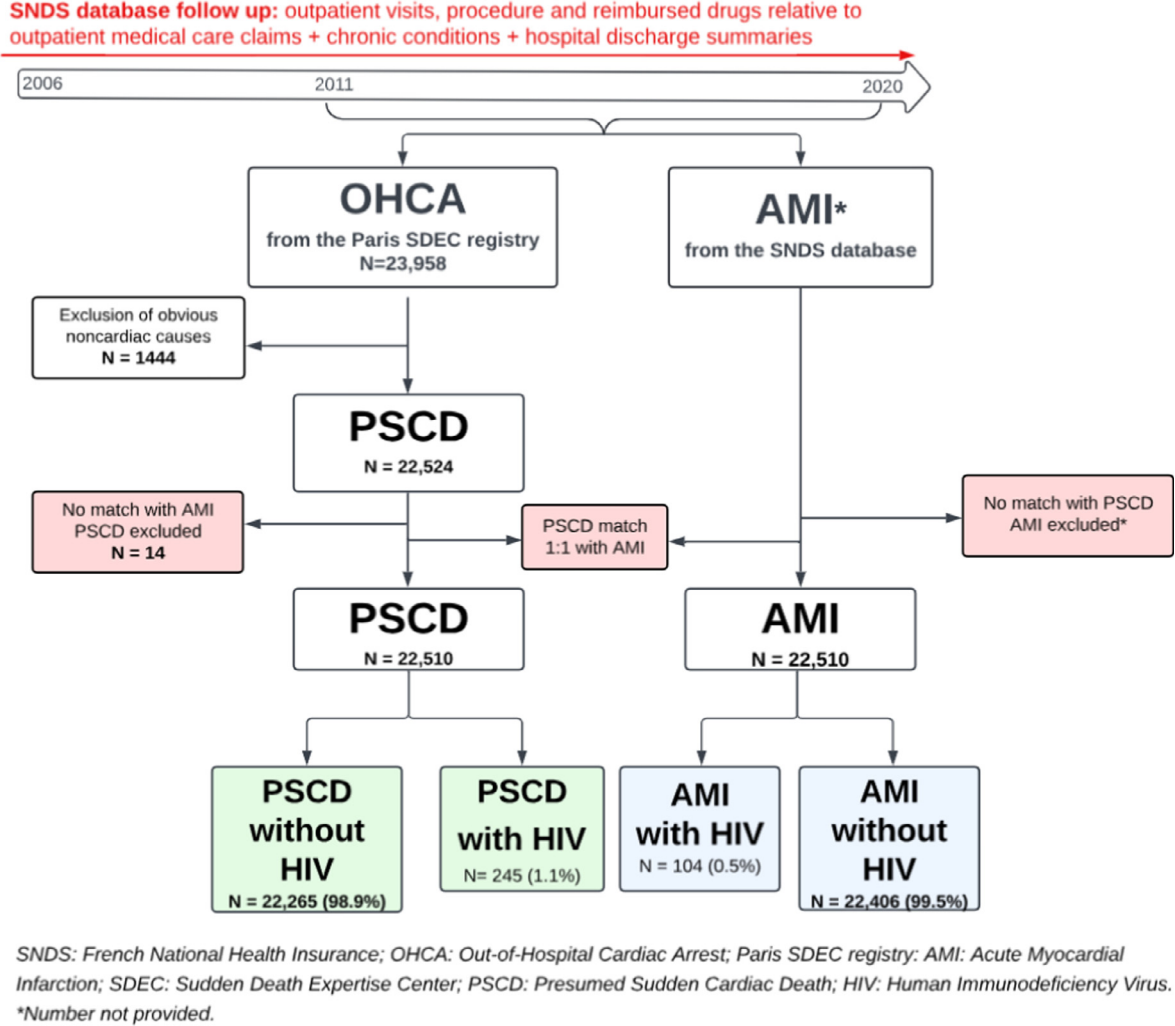
**Flow chart**. *We used the SNDS database to collect medical data spanning the 5 years preceding each PSCD or AMI event, up to the date of occurrence. For the first PSCD patient included in 2011, this retrospective data collection began in 2006.*

In this study, all consecutive cases of unexpected out-of-hospital cardiac arrest in patients 18 years of age or older in the Paris SDEC registry between 2011 and 2020 were included (Fig. 1). PSCD is defined as sudden death presumed to be of cardiac cause that occurs within 1 h of onset of symptoms when witnessed and within 24 h of last being seen alive when unwitnessed; therefore, out-of-hospital cardiac arrest cases due to external causes were excluded from the analyses.^26^ Each case was reviewed separately by two investigators to ensure accuracy of classification. This database followed strict internal and external quality assurance protocols, as previously described.^20^

#### Acute myocardial infarction ascertainment

The SNDS database allowed the identification of patients 18 years of age or older, having an AMI between 2011 and 2020 (Fig. 1, Supplementary Table 1). The accuracy of classification was verified, and several quality assessments were performed using the Healthcare Expenditures and Conditions Mapping (HECM) algorithm, as previously described.^25^, ^27^, ^28^, ^29^ After verification that there are no duplicates in the 2 populations, PSCD patients from the Paris SDEC registry were matched 1:1 on age, sex and department of residence with hospitalized AMI controls from the SNDS database, thus forming the study population.^20^ Only AMI patients matched with PSCD were included; all other AMI patients were excluded. We obtained clearance from the appropriate institutional review boards for the analysis (Institut des Données de Santé, approval N°183, 2016; CCTIRS approval N°12-336, 2016; CNIL authorization DR-2016–401, 2016). This study was conducted in compliance with Strengthening the Reporting of Observational Studies in Epidemiology (STROBE) guidelines for observational studies (Supplementary Table 2).^30^

#### HIV status

HIV patients were identified in the study population from 5 years prior to the occurrence of PSCD or AMI until the event, using the HECM algorithm developed by the French National Health Insurance.^28^, ^29^, ^31^ The earliest possible inclusion date for the HIV population was in 2006, and the latest was in 2020. These patients had more than one reimbursement for anti-retroviral treatment based on an appropriate ATC Classification System code or the presence of an appropriate ICD-10 code before the occurrence of PSCD or AMI (Supplementary Table 1).

### Patient characteristics

Among 22,510 PSCD and 22,510 AMI patients, characteristics including sociodemographic data (age at PSCD or AMI occurrence, sex and complementary universal health insurance (free access to healthcare for people with an annual income <50 % of the poverty threshold)), evidence of cardiovascular diseases (CVD), risk factors and comorbidities (smoking abuse, obesity, dyslipidaemia, diabetes, hypertension, acute stroke, chronic pulmonary disease, chronic kidney disease, active cancer, depressive episode, mood disorders excluding depressive episode, drug use and alcohol abuse), and treatments were recorded up to 5-years prior to the event (Table 1, Supplementary Table 1).

**Table 1:** Patients’ characteristics.

	**Presumed Sudden Cardiac Death**	**Acute Myocardial Infarction**
**N**	22,510	22,510
**HIV, n (%)**	245 (1.1)	104 (0.5)
**SOCIODEMOGRAPHIC CHARACTERISTICS**		
**Men, n (%)**	13,588 (60.4)	13,588 (60.4)
**Age, y, mean (SD)**	71.4 (16.8)	71.5 (16.5)
**Complementary universal health insurance, n (%)**	1377 (6.1)	1091 (4.8)
**CARDIOVASCULAR DISEASES**		
**Cardiac arrhythmia and conduction disorders, n (%)**	5065 (22.5)	3263 (14.5)
**Valvular diseases, n (%)**	1720 (7.6)	1062 (4.7)
**Heart failure, n (%)**	4389 (19.5)	2306 (10.2)
**Pulmonary embolism, n (%)**	1000 (4.4)	383 (1.7)
**Peripheral arterial diseases, n (%)**	2445 (10.9)	1930 (8.6)
**RISK FACTORS AND COMORBIDITIES**		
**Smoking abuse, n (%)**	2761 (12.3)	1764 (7.8)
**Obesity, n (%)**	2278 (10.1)	1682 (7.5)
**Dyslipidaemia, n (%)**	3517 (15.6)	3662 (16.3)
**Diabetes, n (%)**	6000 (26.7)	5867 (26.1)
**Hypertension, n (%)**	9531 (42.3)	8253 (36.7)
**Acute stroke, n (%)**	1203 (5.3)	641 (2.8)
**Chronic pulmonary disease, n (%)**	3727 (16.6)	1758 (7.8)
**Chronic kidney disease, n (%)**	3267 (14.5)	2014 (8.9)
**Active cancer, n (%)**	4694 (20.9)	2383 (10.6)
**Mood disorders excluding depressive episode, n (%)**	591 (2.6)	245 (1.1)
**Depressive episode, n (%)**	1676 (7.4)	779 (3.5)
**Alcohol abuse, n (%)**	2589 (11.5)	870 (3.9)
**Drug use, n (%)**	453 (2.0)	107 (0.5)
**TREATMENTS BEFORE THE EVENT**		
**Agents acting on the renin-angiotensin system, n (%)**	12,552 (55.8)	12,668 (56.3)
**Diuretics, n (%)**	11,902 (52.9)	10,466 (46.5)
**Beta blocking agents, n (%)**	9523 (42.3)	9527 (42.3)
**Calcium channel blockers, n (%)**	8894 (39.5)	9120 (40.5)
**Statins, n (%)**	9734 (43.2)	11,242 (49.9)
**Aspirin, n (%)**	9962 (44.3)	11,188 (49.7)
**Other antiplatelet agents than Aspirin, n (%)**	3446 (15.3)	4985 (22.1)
**Oral anticoagulants, n (%)**	5296 (23.5)	3467 (15.4)
**Antiarrhythmic agents, n (%)**	3219 (14.3)	2129 (9.5)

HIV: Human Immunodeficiency Virus; SD: standard deviation.

### Statistical analysis

Continuous data are presented as mean (SD) and categorical data are presented as n(%). We used a multivariable logistic regression model to investigate the association between HIV infection and the type of cardiac event. The binary outcome variable was coded as 1 for PSCD and 0 for AMI. The main exposure was HIV status (positive vs negative). The main exposure was HIV status (positive vs negative). We adjusted for potential confounders, including age, sex, smoking abuse, mood disorders excluding depressive episode, drug use and alcohol abuse, identified a priori based on clinical knowledge and a directed acyclic graph (DAG). Odds-ratio (OR) and their 95 % confidence intervals (95 % CI) were calculated with adjustment for potential confounding factors. To assess the robustness of our results to potential misclassification of binary covariates due to under-recording in electronic health records, we performed a sensitivity analysis with a scenario-based multiple imputation procedure.^32^ Another sensitivity analysis was conducted, excluding PSCD patients with a history of AMI. P values less than 0.05 were considered statistically significant. All statistical analyses were performed using the open-source Python 3.8.0 software package.

## Results

### Baseline characteristics

This study included 22,510 PSCD patients (60 % men, mean age 71 (17) years) who were matched with 22,510 AMI controls (60 % men, mean age 72 (17) years) (Fig. 1). Among these patients, 245 (1 %) had a positive HIV status preceding the PSCD and 104 (1 %) preceding the AMI (Table 1). PSCD patients with HIV-positive status were younger than PSCD patients with HIV-negative status (56 (11) vs. 71 (17)) and AMI patients with HIV-positive status were younger than AMI patients with HIV-negative status (57 (10) vs. 72 (17)). HIV patients were also significantly more frequently men than uninfected patients in the PSCD and AMI populations (78 % vs. 60 % and 88 % vs. 60 % respectively).

The baseline characteristics of HIV patients in the PSCD and AMI populations are presented in Table 2.

**Table 2:** Characteristics of patients with human immunodeficiency virus.

	**HIV + Presumed Sudden Cardiac Death**	**HIV + Acute Myocardial Infarction**
**N**	245	104
**SOCIODEMOGRAPHIC CHARACTERISTICS**		
**Men, n (%)**	192 (78.4)	91 (87.5)
**Age, y, mean (SD)**	55.6 (10.7)	57.4 (10.0)
**Complementary universal health insurance, n (%)**	18 (7.3)	3 (2.9)
**CARDIOVASCULAR DISEASES**		
**Cardiac arrhythmia and conduction disorders, n (%)**	33 (13.5)	7 (6.7)
**Valvular diseases, n (%)**	11 (4.5)	3 (2.9)
**Heart failure, n (%)**	41 (16.7)	12 (11.5)
**Pulmonary embolism, n (%)**	16 (6.5)	1 (1.0)
**Peripheral arterial diseases, n (%)**	31 (12.7)	13 (12.5)
**RISK FACTORS AND COMORBIDITIES**		
**Smoking abuse, n (%)**	83 (33.9)	20 (19.2)
**Obesity, n (%)**	31 (12.7)	10 (9.6)
**Dyslipidaemia, n (%)**	49 (20.0)	25 (24.0)
**Diabetes, n (%)**	61 (24.9)	25 (24.0)
**Hypertension, n (%)**	98 (40.0)	31 (29.8)
**Acute stroke, n (%)**	22 (9.0)	5 (4.8)
**Chronic pulmonary disease, n (%)**	59 (24.1)	12 (11.5)
**Chronic kidney disease, n (%)**	54 (22.0)	18 (17.3)
**Active cancer, n (%)**	79 (32.2)	13 (12.5)
**Mood disorders excluding depressive episode, n (%)**	25 (10.2)	3 (2.9)
**Depressive episode, n (%)**	58 (23.7)	7 (6.7)
**Alcohol abuse, n (%)**	77 (31.4)	9 (8.7)
**Drug use, n (%)**	63 (25.7)	5 (4.8)
**TREATMENTS BEFORE THE EVENT**		
**Agents acting on the renin-angiotensin system, n (%)**	112 (45.7)	45 (43.3)
**Diuretics, n (%)**	101 (41.2)	29 (27.9)
**Beta blocking agents, n (%)**	99 (40.4)	37 (35.6)
**Calcium channel blockers, n (%)**	76 (31.0)	28 (26.9)
**Statins, n (%)**	97 (39.6)	59 (56.7)
**Aspirin, n (%)**	104 (42.4)	52 (50.0)
**Other antiplatelet agents, n (%)**	33 (13.5)	27 (26.0)
**Oral anticoagulants, n (%)**	33 (13.5)	8 (7.7)
**Antiarrhythmic agents, n (%)**	9 (3.7)	1 (1.0)

HIV: Human Immunodeficiency Virus; SD: standard deviation.

### Logistic regression analyses

**Fig. 2:**
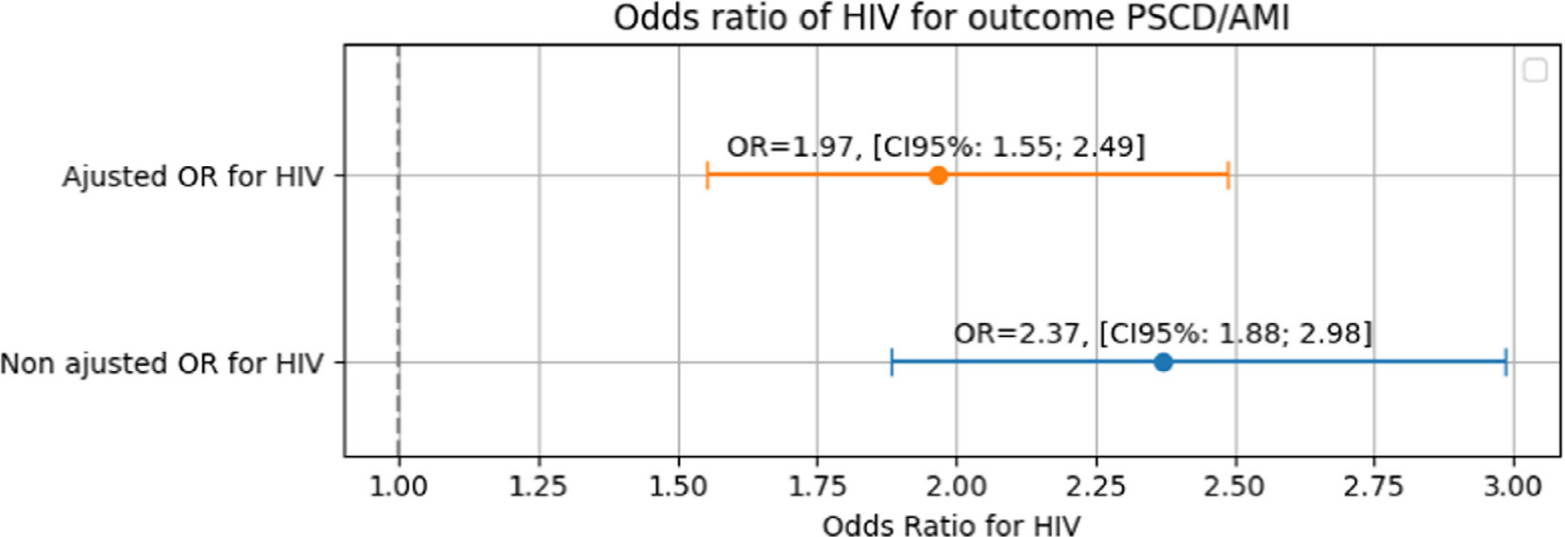
**Logistic regression evaluating the association between HIV and PSCD compared to HIV and AMI**. *HIV: Human Immunodeficiency Virus; PSCD: Presumed Sudden Cardiac Death; AMI: Acute Myocardial Infarction; OR: Odds-Ratio; CI: Confidence Interval. Odds-ratio for HIV was adjusted for potential confounders, including age, sex, smoking abuse, mood disorders excluding depressive episode, drug use and alcohol abuse, identified a priori based on clinical knowledge and a directed acyclic graph (DAG).*

The odds of PSCD was 97 % higher than the odds of AMI in HIV patients after adjusting for potential confounders, including age, sex, smoking abuse, mood disorders excluding depressive episode, drug use and alcohol abuse, identified a priori based on clinical knowledge and a directed acyclic graph (DAG), with an adjusted odds-ratio, 1.97; 95 % CI: 1.55–2.49 (Supplementary Fig. 1, Fig. 2).

Four covariates in our logistic regression model—mood disorders excluding depressive episode, smoking abuse, drug use, and alcohol abuse—were identified as potentially under-recorded and were imputed. Therefore, a sensitivity analysis using a scenario-based multiple imputation procedure yielded results consistent with the main analysis (Supplementary Table 3). Another sensitivity analysis excluding 978 PSCD patients with an AMI history showed a similar result as the main analysis. The odds of PSCD was 64 % higher than the odds of AMI in HIV patients with an adjusted odds-ratio, 1.64; 95 % CI: 1.21 to 2.22 after adjusting for age, sex, smoking abuse, mood disorders excluding depressive episode, drug use and alcohol abuse.

## Discussion

Our findings indicate that, among people living with HIV, the odds of PSCD exceeds that of AMI. This elevated odds highlights the importance of early identification of HIV subjects who may be at increased risk of sudden cardiac death, in order to implement timely and targeted preventive strategies. To our knowledge, the approach of selecting patients with AMI as controls was a novel one in this space and the association between HIV and PSCD compared to HIV and AMI was not described yet.

The SNDS, officially established in 2016, builds on earlier systems, particularly the SNIIRAM, created in 1999 to collect nationwide health insurance reimbursement data. Over time, the SNDS expanded to include hospital records (PMSI), causes of death, disability data, and, by 2006, risk factors, comorbidities, and treatments identified through ICD-10 and ATC codes. For the Paris SDEC Registry, the first patient was included in 2011, allowing for a 5-year retrospective review of medical history. This 5-year look-back period was consistently applied to all patients in the study to ensure standardized inclusion of clinical data.

Myocardial dysfunction has been known a complication of HIV since 1980*.*^33^, ^34^ A 2018 meta-analysis, which included 793,635 individuals living with HIV revealed that the global burden of HIV-associated CVD tripled over the past 2 decades and the relative risk of AMI was 1.5- to 2-fold greater for HIV patients compared with uninfected individuals.^3^ The latter years, improvements due to easy access to modern combination ART regimens in conjunction with aggressive primary and secondary prevention of CVD may have had a significant impact in reducing coronary heart disease among HIV-infected patients.^35^ Recent studies reported that smoking cessation could reduce AMI risk in HIV-positive individuals.^36^, ^37^

In contrast to our approach of selecting patients with AMI as controls, all studies that evaluated the association between HIV and PSCD used the general population as a control group and all of them except Freiberg study (2021) reported a small number of PSCD.^12^, ^13^, ^14^, ^15^, ^16^ HIV patients had a fourfold higher incidence of PSCD compared to the general or heart failure (HF) population.^12^, ^13^, ^14^ Our findings align with this overall trend, further supporting the elevated risk of PSCD in people with HIV. However, by using AMI patients as a comparator group, our study offers a more rigorous assessment of this association, highlighting a link between HIV and PSCD that extends beyond general cardiovascular risk.

Although the prevalence of HIV infection is considerably lower than that of traditional CVD risk factors, our study highlights that HIV infection may confer a disproportionately high risk of PSCD compared to AMI. This finding is particularly concerning and suggests that mechanisms beyond conventional risk factors may be at play in HIV-positive individuals. While the exact pathways remain incompletely understood, previous studies have pointed to a complex interplay of factors, including HIV itself, antiretroviral therapy (ART), and traditional CVD risk factors.^3^, ^38^

Our results align with prior evidence suggesting that HIV-related immunologic and inflammatory processes—as well as metabolic disturbances such as dyslipidaemia, insulin resistance, and substance abuse—may contribute to cardiovascular complications in people living with HIV.^39^, ^40^ Of note, ventricular tachyarrhythmias, which are often linked to coronary artery disease, can be the underlying cause of PSCD.^41^ A recent study further supports this possibility by demonstrating increased ventricular repolarization lability and autonomic dysfunction in HIV-positive individuals, potentially explaining their heightened susceptibility to sudden cardiac death.^42^ The increased odds of PSCD compared to AMI in HIV patients, observed in our study, supports the existence of a possibility of a greater susceptibility to ventricular fibrillation in this population The over-risk of PSCD compared to AMI in HIV patients, observed in our study, supports the existence of a possibility of a greater susceptibility to ventricular fibrillation in this population. This is in line with a recent postmortem study showing higher arrhythmic PSCD incidence in HIV-positive individuals (25.0 per 100,000 person-years) compared to those without known HIV infection (13.3 per 100,000 person-years). Additionally, histologic examination revealed greater levels of interstitial myocardial fibrosis in HIV-positive decedents, suggesting a possible structural substrate for arrhythmias.^15^ HF may also play a role in PSCD among HIV-positive individuals. Prior studies have demonstrated an increased risk of both HF with reduced and preserved ejection fraction in this population.^15^, ^43^ Furthermore, other structural heart diseases, such as HIV-associated cardiomyopathy, and rarer causes like channelopathies, must be considered.^44^, ^45^ *Importantly, not all PSCDs in HIV-positive individuals are of cardiac origin. The same postmortem study reported that approximately one-third of PSCDs in this group were attributable to occult drug overdose, emphasizing the multifactorial nature of sudden death in this population.*^15^ In our study, drug use was more frequent among PSCD patients with HIV-positive status compared to those with HIV-negative status (26 % vs. 2 %, respectively).

Taken together, our findings underscore the need for broader clinical awareness of PSCD risk in people living with HIV and highlight the importance of further research to disentangle the contributing biological and social determinants. In this context, future prospective studies are essential to deepen our understanding of the complex interplay between modifiable risk factors and sudden cardiac death in HIV-positive patients, and to determine whether the pathological electrophysiological processes underlying arrhythmias can be reversed through targeted interventions.^46^

Our study has some limitations. First, the number of HIV patients was low. However, the use of a large population-based registry allowed to observe 22,510 PSCD and 22,510 AMI patients derived from the comprehensive French healthcare databases. It represents a unique opportunity to overcome the issue of the small number of PSCD events in cohorts of HIV patients. Second, our results need to be interpreted with caution and our novel findings require replication. the generalizability of our findings to a broader geographical context may be limited because of the differences among countries with respect to the healthcare system, lifestyle, environment, and genetics.^47^ Third, the indices of advanced-stage HIV infections such as viral loads and CD4 counts, and some antiretroviral therapies, which are known to be independently associated with increased risk of CVD, were unfortunately not available in our study. In addition, pre-exposure prophylaxis (PrEP) patients were not excluded from the HIV-positive population, which may lead to an underestimation of the true association. Moreover, we were also unable to evaluate adherence to treatment of HIV patients. Fourth, we acknowledge that none of our PSCD had postmortem confirmation reflecting the reality that autopsy of these out-of-hospital deaths in France is rare. In the absence of autopsy for the PSCD cases, we cannot know the underlying etiology (ischemic, arrhythmic or other causes of death). Fifth, in our study, drug use was more frequent among PSCD patients with HIV-positive status compared to those with HIV-negative status (26 % vs. 2 %, respectively). This difference observed in drug use, as well as in other forms of substance abuse and comorbidities, may partly reflect more frequent healthcare contact and better documentation among HIV-positive patients, while HIV-negative individuals might be underdiagnosed due to less medical engagement.Finally, with regard to missing data, missing cases were unlikely for PSCD and HIV due to robust verification and systematic electronic data collection, respectively. For AMI, missing cases were not a concern since the analysis focused on 1:1 matching with the Paris SDEC registry rather than exhaustive case capture. To address the potential impact of systematic under-recording in electronic health records, we conducted a sensitivity analysis applying a multiple imputation approach. This provided reassurance that potential misclassification in our data had minimal impact on the estimated association between exposure and outcome. Given that sudden cardiac death can occur as a direct complication of AMI, we also conducted a sensitivity analysis to evaluate the association between HIV status and PSCD among individuals without a known history of AMI. This approach aimed to better isolate cases of PSCD that may be independent of overt ischemic events and to explore whether HIV infection contributes to arrhythmic death through non-ischemic pathways. We also acknowledge that in some patients, sudden death may represent the first manifestation of AMI, which complicates the interpretation. However, by excluding individuals with documented AMI, our analysis strengthens the case for a probably distinct, potentially HIV-related susceptibility to primary arrhythmic events, independent of traditional coronary disease presentations.

Our findings strongly suggest a significant association between HIV status and PSCD, also among patients without a history of AMI. The underlying mechanisms still remain incompletely defined and further studies are needed.

## Data availability

This study was performed using the PMSI (Programme de Médicalisation des Systèmes d’Information) French national database and restrictions apply to the availability of these data, which were used under license for this study. The data will be shared on reasonable request to the corresponding author.

## CRediT authorship contribution statement

**Patricia Jabre:** Writing – original draft, Data curation, Conceptualization. **Richard Chocron:** Writing – review & editing, Formal analysis. **Thomas Laurenceau:** Writing – review & editing, Formal analysis. **Marion Chabrol:** Writing – review & editing, Formal analysis. **Ugo Meli:** Writing – review & editing, Formal analysis. **Younès Youssfi:** Writing – review & editing, Formal analysis. **Marie Cécile Perier:** Writing – review & editing, Data curation. **Wulfran Bougouin:** Writing – review & editing, Conceptualization. **Frankie Beganton:** Writing – review & editing, Data curation. **Thomas Loeb:** Writing – review & editing, Data curation. **François Revaux:** Writing – review & editing, Data curation. **Daniel Jost:** Writing – review & editing, Data curation. **Alain Cariou:** Writing – review & editing, Conceptualization. **Jean-Philippe Empana:** Writing – review & editing, Conceptualization. **Frédéric Adnet:** Writing – review & editing, Conceptualization. **Xavier Jouven:** Writing – original draft, Supervision, Conceptualization.

## Funding

No funding sources for this study.

## Declaration of competing interest

The authors declare that they have no known competing financial interests or personal relationships that could have appeared to influence the work reported in this paper.
