# Supplementary material for: Association of human immunodeficiency virus with acute myocardial infarction and presumed sudden cardiac death
Source: Resusc Plus. 2025 Jul 19;25:101035. doi: 10.1016/j.resplu.2025.101035 (PMC12329076; doi:10.1016/j.resplu.2025.101035)
Supplement: Supplementary Data 1 [file mmc1.docx]

|  |
| --- |
|  |
| **Supplement**  **Figure 1: Directed Acyclic Graphs (DAG) for understanding of adjustment process, using the online software**[**https://www.dagitty.net/**](https://www.dagitty.net/)**.** |


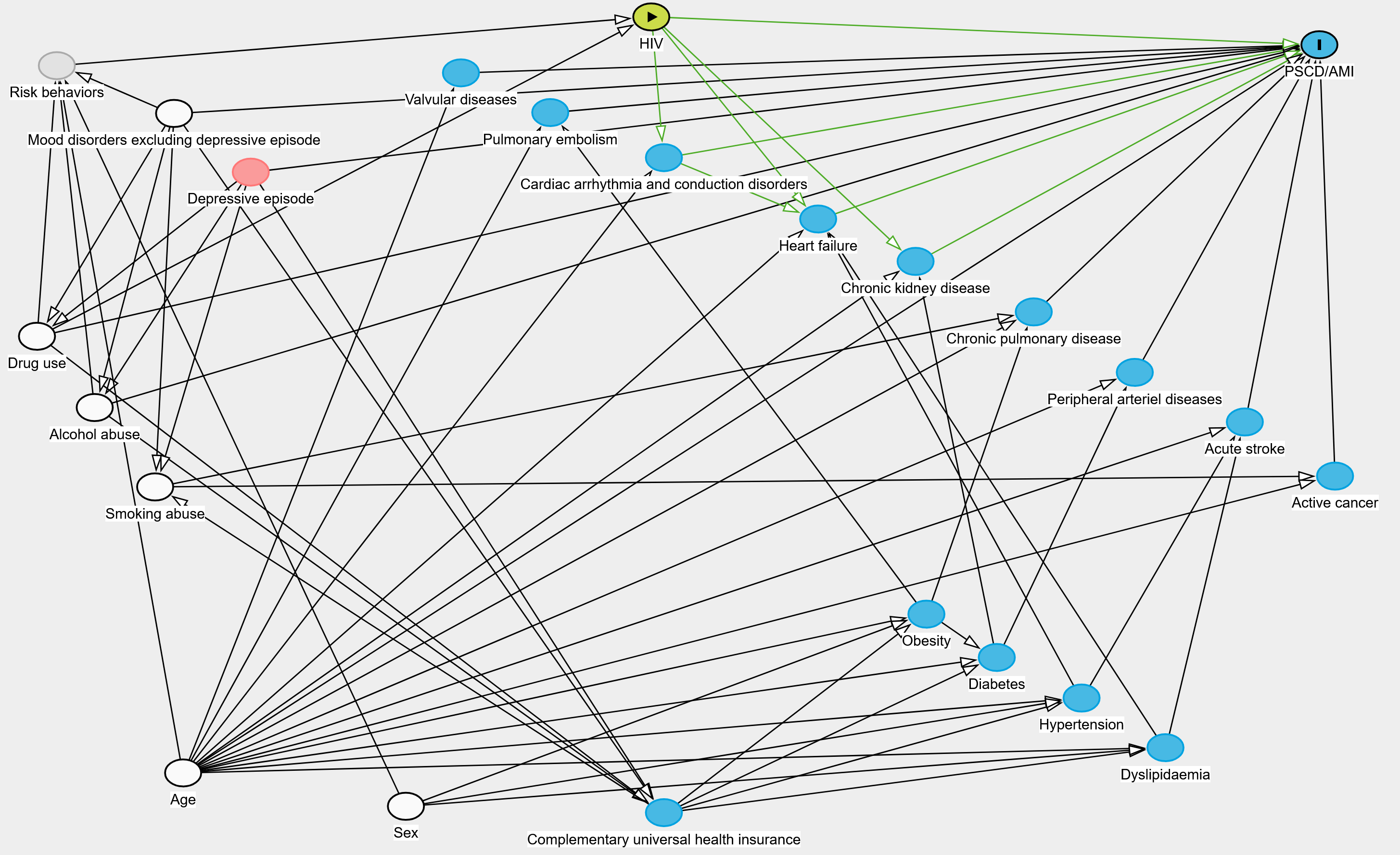


*HIV: Human Immunodeficiency Virus; PSCD: Presumed Sudden Cardiac Death; AMI: Acute Myocardial Infarction.*
