# Supplementary material for: Association of human immunodeficiency virus with acute myocardial infarction and presumed sudden cardiac death
Source: Resusc Plus. 2025 Jul 19;25:101035. doi: 10.1016/j.resplu.2025.101035 (PMC12329076; doi:10.1016/j.resplu.2025.101035)
Supplement: Supplementary Data 2 [file mmc2.docx]

|  | |
| --- | --- |
|  | |
| **Supplement**  **Table 1: International Classification of Diseases, 10th revision (ICD-10) codes used for each diagnosis and Anatomical Therapeutic Chemical (ATC) classification codes used for each treatment** | |
| **Etiologies and Covariates** | **Definition** |
| **Myocardial infarction** | ICD-10: I21, I22, I23 |
| **Hunan Immunodeficiency Virus** | ICD-10: B20, B21, B22, B23, B24, Z21  ATC: J05AF01, J05AF02, J05AF03, J05AF04, J05AF05, J05AF06, J05AF07, J05AF08, J05AF09, J05AG01, J05AG03, J05AG04, J05AG05, J05AR01, J05AR02, J05AR03, J05AR04, J05AR06, J05AR08, J05AR09, J05AR10, J05AE01, J05AE02, J05AE03, J05AE04, J05AE05, J05AE06, J05AE07, J05AE08, J05AE09, J05AE10, J05AX07, J05AX08, J05AX09 |
| **Cardiovascular diseases** | |
| **Cardiac arrhytmias and conduction disorders** | ICD-10: I44, I45, I47, I48, I49 |
| **Valvular diseases** | ICD-10: I05, I06, I07, I08, I34, I35, I36, I37, I38, I39 |
| **Heart failure** | ICD-10: I110, I130, I132, I139, I50, J81, K761 |
| **Pulmonary embolism** | ICD-10: I26, I270 |
| **Peripheric arterial diseases** | ICD-10: I70, I71, I72, I73, I74, I77, I78, I79 |
| **Risk factors and comorbidities** | |
| **Smoking abuse** | ICD-10: F17, T652, Z587, Z716, Z720 |
| **Obesity** | ICD-10: E66, T855 |
| **Dyslipidaemia** | ICD-10: E78 |
| **Diabetes** | ICD-10: E10, E11, E12, E13, E14, G590, G632, G730, G990, H280, H360, M142, N083 |
| **Hypertension** | ICD-10: I10, I11, I12, I13, I15, I674 |
| **Acute stroke** | ICD-10: I60, I61, I62, I63, I64 |
| **Chronic pulmonary disease** | ICD-10: I278, I279, J40, J41, J42, J43, J44, J45, J46, J47, J60, J61, J62, J63, J64, J65, J66, J67, J684, J701, J703, J961 |
| **Chronic kidney disease** | ICD-10: E102, E112, E132, E142, I12, I131, I132, N032, N033, N034, N035, N036, N037, N052, N053, N054, N055, N056, N057, N083, N18, N19, N250, Z49, Z940, Z992 |
| **Active cancer** | ICD-10: C00-C97, D00, D01, D02, D03, D04, D05, D06, D07, D08, D09, D37, D38, D39, D40, D41, D42, D43, D44, D45, D46, D47, D48, D49 |
| **Mood disorders excluding depressive episode** | ICD-10: F20.0, F20.1, F20.2, F20.3, F20.5, F20.81, F20.89, F20.9, F22, F23, F24, F25.0, F25.1, F25.8, F25.9, F28, F29, F30, F31, F32.4, F32.5, F33.4, F34.8, F34.9, F39, F84.3 |
| **Depressive episode** | ICD-10: F32.0, F32.1, F32.2, F32.3, F32.8, F32.9, F33.0, F33.1, F33.2, F33.3, F33.8, F33.9, F34.1 |
| **Alcohol abuse** | ICD-10: E244, F10, I426, G312, G621, G721, K292, K70, K852, K860, T51, X65, Y573, Y90, Y919, Z502, Z714, Z721 |
| **Drug use** | ICD-10: F11, F12, F13, F14, F16, F19, R78, T40, T436, Z503, Z715, Z722, Z8641 |
| **Treatments** | |
| **Agents acting on the renin-angiotensin system** | ATC: C09A, C09B, C09C, C09D, C09XA02 |
| **Diuretics** | ATC: C02LA01, C03, C07BA02, C07BB, C07CA03, C07DA06, C08GA02, C09BA, C09DA, C09XA52, S01EC01 |
| **Βeta blocking agents** | ATC: C07 |
| **Calcium channel blockers** | ATC: C07FB, C08, C09BB, C09DB, C10BX03 |
| **Statins** | ATC: C10AA, C10BA, C10BX |
| **Aspirin** | ATC: B01AC06, B01AC08, B01AC30, C10BX02, N02BA01 |
| **Other antiplatelet agents than Aspirin** | ATC: B01AC |
| **Oral anticoagulants** | ATC: B01AA, B01AE, B01AF, B01AX |
| **Antiarrhythmic agents** | ATC: C01B |
