# Supplementary material for: Association of human immunodeficiency virus with acute myocardial infarction and presumed sudden cardiac death
Source: Resusc Plus. 2025 Jul 19;25:101035. doi: 10.1016/j.resplu.2025.101035 (PMC12329076; doi:10.1016/j.resplu.2025.101035)
Supplement: Supplementary Data 4 [file mmc4.docx]

**Supplement**

**Table 2: Logistic regression evaluating the association between HIV and PSCD compared to HIV and AMI after multiple imputation of missing data**

| **Variables** | **OR** | **95%CI** |
| --- | --- | --- |
| HIV | 1.99 | 1.57-2.52 |
| Age | 1.00 | 1.00-1.00 |
| Male Sex | 0.99 | 0.95-1.03 |
| Mood disorders excluding depressive episode | 1.06 | 1.01-1.13 |
| Smoking abuse | 1.15 | 1.13-1.18 |
| Drug use | 1.06 | 1.02-1.12 |
| Alcohol abuse | 1.02 | 1.02-1.02 |

*HIV: Human Immunodeficiency Virus; PSCD: Presumed Sudden Cardiac Death; AMI: Acute Myocardial Infarction; OR: Odds-Ratio; CI: Confidence Interval.*
